# Silicon Nanostructures Produced by Modified MacEtch Method for Antireflective Si Surface

**DOI:** 10.1186/s11671-017-1886-2

**Published:** 2017-02-10

**Authors:** Stepan Nichkalo, Anatoly Druzhinin, Anatoliy Evtukh, Oleg Bratus’, Olga Steblova

**Affiliations:** 1 Lviv Polytechnic National University, 12 S. Bandera Str., 79013 Lviv, Ukraine; 2 Institute of Semiconductor Physics, National Academy of Sciences of Ukraine, 41 pr. Nauki, 03028 Kyiv, Ukraine; 3 Institute of High Technologies, Taras Shevchenko National University of Kyiv, 4-g prosp. Glushkova, 03022 Kyiv, Ukraine

**Keywords:** Silicon, Nanowires, Nanopores, Metal-assisted chemical etching, Coagulation, Absorption, Reflectance

## Abstract

This work pertains to the method for modification of silicon (Si) wafer morphology by metal-assisted chemical etching (MacEtch) technique suitable for fabrication of antireflective Si surfaces. For this purpose, we made different Au catalyst patterns on the surface of Si substrate. This modification allowed to obtain the close-packed Au nanodrop (ND) pattern that generates the nanowires (NWs) and the well-separated Au NDs, which induce the nanopore (NP) formation. The antireflective properties of these structures in comparison with NWs produced by the conventional Ag-MacEtch method were analysed. The total surface reflectance of 1~7% for SiNWs and ~17% for SiNPs was observed over the entire Si-absorbing region. Moreover, SiNWs prepared by Au-MacEtch demonstrate better antireflective properties in contrast to those formed by conventional Ag-assisted chemical etching. So, the use of SiNWs produced by the modified Au-MacEtch method as the antireflective material is favored over those prepared by Ag-MacEtch due to their higher light absorption and lower reflectance. The possible reason of these findings is discussed.

## Background

Photovoltaic manufacturing is one of the most perspective branches of modern industry, which develops intensively and demonstrates larger percentage of electrical power production growth [1]. To achieve a high-efficiency Si solar cell (SC), antireflective layers/structures are inevitably necessary [2–6]. Fine surface structures, comprising features on the nanometer scale, can provide excellent antireflective performance [3–6]. In this regard, a recently developed metal-assisted chemical etching is a method that produces anisotropic high aspect ratio nanowires (NWs), which reduce optical loss, enhance optical absorption, and improve carrier extraction for high performance and low-cost solar cells [7–13]. In addition to NWs, the nanoporous Si surfaces prepared by MacEtch demostrate good antireflective properties also. In particular, Peng et al. showed that the efficiency of nanopore-based SCs can be as high as 9.51% [14]. In their work [15], authors have shown that nanoporous structures require several times less Si by mass to obtain the same ultimate efficiency as a standard Si wafer. In our previous works [16–19], we showed that micro- and nanotexturization of the Si wafer by chemical vapor deposition (CVD)-grown SiNWs and MacEtch-ed nanopores enhance an optical absorption spectra. However, because of random distribution and non-controllable orientation of SiNWs, as a result of vapor-liquid-solid crystal growth [20], the efficiency of such SC was still low.

As it is well known, NWs or nanopores (NPs) can be formed from different noble metal-catalyst patterns, e.g., Ag nanoparticle network is self-generated from AgNO_3_ solution [9, 21–23] or an Au thin film thermally deposited on Si substrate [24]. The solution-based patterning is simple and less expensive approach but doesn't provide a good control over the produced feature size and shape [25]. Moreover, the etch rate is ~10 times slower than that of typical thin film catalyzed MacEtch [26].

The aim of this work is the modification of Si wafer morphology by the MacEtch method for fabrication of antireflective Si surfaces. The technological features of MacEtch producing SiNWs and SiNPs with the right size and density were also considered and analyzed. Taking into account the fact that the distance between metal catalyst particles strongly influences the morphology of the etched structures [27, 28], we proposed to form two different Au-catalyst patterns on the surface of Si substrate. This modification allowed to obtain the close-packed Au nanodrop (ND) pattern that generates the NWs and the well-separated Au NDs, which induce the NP formation. The antireflective properties of these structures in comparison with NWs produced by the conventional Ag-MacEtch method were also analyzed.

## Methods

For obtaining the nanostructured Si surfaces, the p-type Si wafers with crystallographic orientation (100) and resistivity 10 Ω × cm were used. The wafers were cut into samples of 2 × 2 cm^2^. The chemical cleaning of Si wafer samples was conducted according to the RCA procedure [29], which is used in the semiconductor industry for removing organic and metal contaminants. It included at the first phase the treatment in a mixture of water, hydrogen peroxide (35%), and ammonium hydroxide (27%) H_2_O/H_2_O_2_/NH_4_OH at a ratio of 5:1:1. The cleaning process was carried out at 75 °C for 10 min followed by rinsing in deionized (DI) water and drying. Afterwards, the specimens were immersed in solution consisting of HF (49%) and H_2_O (1:10) for 5 min to remove the layer of native oxide SiO_2_.

The catalyst pattern formation on Si wafer was realized through two different deposition approaches, namely, (i) the self-generation of dendrite-like Ag network from AgNO_3_ solution (for Ag-MacEtch) and (ii) the evaporation of an Au thin film (for Au-MacEtch).

In the first approach, Ag nanoparticles were deposited on Si surface from AgNO_3_/HF (0.02/4.6 M) solution for 2 min at room temperature. The chemical etching of Si samples, coated by Ag nanoparticles, was performed at room temperature in HF/H_2_O_2_ (4.6/0.15 M) system for 3 min.

**Fig. 1:**
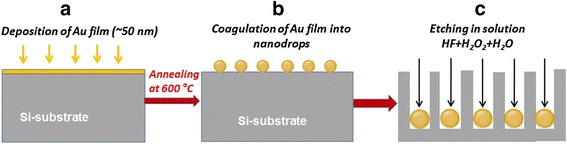
Schematic view of the SiNW formation on Si substrate by the modified Au-MacEtch process, which is divided in three steps: **a** thermal vacuum deposition of Au thin film on Si substrate; **b** thermal annealing of gold-coated Si substrate for the coagulation of deposited Au thin film into nanodrops; **c** etching of the prepared samples in HF/H_2_O_2_/H_2_O solution

Illustrated in Fig. 1 is the formation process of SiNWs on Si substrate using modified MacEtch, which includes the next steps: thermal vacuum deposition of metal catalyst (Au) on Si substrate (see Fig. 1a), annealing of samples at 600 °C in vacuum chamber for 30 min to coagulation of an Au thin film into the nanodrops (Fig. 1b), and etching of as-prepared samples in the etchant consisting of HF (49%)+H_2_O_2_ (35%)+H_2_O = 4:1:40 for 10–15 min for subsequent NW formation (Fig. 1c).

After chemical treatment, the samples were rinsed several times in DI water and dried. The residual gold particles were removed in a low concentrated aqua regia solution.

The surface morphology of Si samples was examined using a scanning electron microscopy (106I SEM, JEOL JSM-U3 SEM, Hitachi S-4800 SEM). The absorption and reflectance spectra of nanostructured Si surfaces were obtained on Specord Plus and Shimadzu UV-3101PC spectrophotometers.

## Results and Discussion

**Fig. 2:**
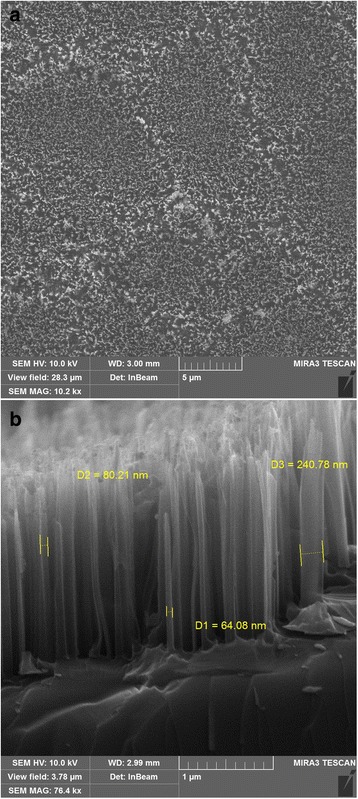
Top view (**a**) and side view (**b**) SEM images of an array of SiNWs, produced by Ag-MacEtch in HF/H_2_O_2_ solution after 3-min treatment

Figure 2 shows the top view (a) and cross-section (b) SEM images of Si substrate after Ag-MacEtch treatment in HF/H_2_O_2_. As a result of etching, the vertically aligned SiNW arrays with diameters ranging from 64 to 240 nm and a height of about 2 μm were formed on Si substrate (Fig. 2b).

**Fig. 3:**
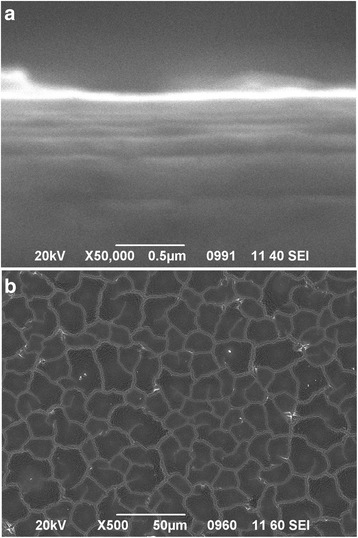
Cross-section (**a**) and top view (**b**) SEM images of Si substrate covered with an Au thin film (50 nm) thermally deposited at 1.5 × 10^−5^ Torr vacuum

Figure 3 shows the cross-section (a) and top view (b) SEM images of Si wafer covered with an Au thin film, which was thermally deposited at 1.5 × 10^−5^ Torr vacuum. The thickness of an Au thin film was determined by a weight, and it was 50 nm thick, as it could be estimated from Fig. 3a. Furthermore, Fig. 3b shows that the film is discontinuous, consisting of isolated islands.

**Fig. 4:**
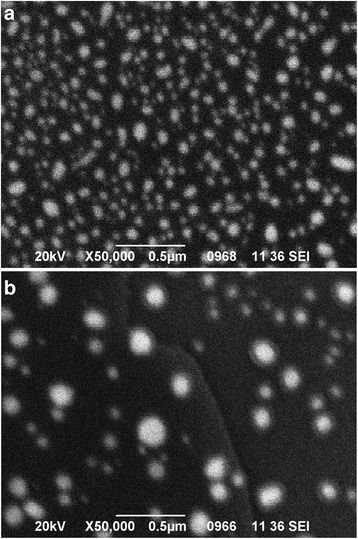
SEM images of close-packed (**a**) and non-close-packed (**b**) Au nanodrops coagulated from a 50-nm thick Au film after thermal annealing of Si substrate at 600 °C for 30 min

The thermal annealing of gold-covered Si specimens at 600 °C for 30 min resulted in the coagulation of an Au thin film into nanodrops. Figure 4a shows that the nanodrops are close-packed with high density and their mean diameter is about 200 nm. As it was mention above, to obtain a nanoporous Si surface, the Au nanodrop catalysts must be well separated on Si substrate. This was achieved by varying the annealing time. Figure 4b shows that the increase of annealing time to 54 min led to the formation of non-close-packed nanodrops with a diameter ranging from 250 up to 1 μm. The observed nanodrop size enlargement coincides with the results obtained by Naydich et al. [30].

**Fig. 5:**
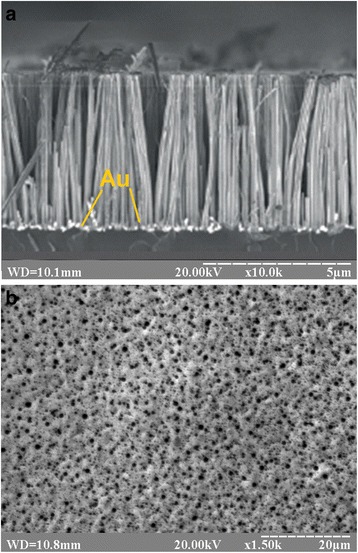
SEM images of SiNWs formed from close-packed Au nanodrops (**a**), and SiNPs formed from non-close-packed Au nanodrops (**b**) after 30 min of etching of Si substrates in HF/H_2_O_2_/H_2_O solution

Shown in Fig. 5a, b are the SEM images of etched Si surface after 30 min treatment in HF/H_2_O_2_/H_2_O (4:1:40) solution. Thus, the pattern from close-packed Au nanodrops induced the formation of vertically aligned SiNWs with an average diameter of about 200 nm (Fig. 5a). In contrast to 2-μm-long SiNWs produced by Ag-MacEtch, the 5-μm-long SiNWs were obtained by the Au-MacEtch method at the same etching time and temperature. The possible explanation for this lies in the effect of thermal annealing on adhesion properties of Si surface, thus providing a high binding energy and good contact at the Au nanodrop/Si surface interface. As a result, the etching starts immediately along the vertical direction. Meanwhile, due to a poor contact between Ag particles precipitated from AgNO_3_ solution and Si surface, the lateral etching of the latter may occur at the initial stage of Ag-MacEtch and the decreasing of total etch rate, as a consequence.

Another nanostructured Si surface predicted the use of well-separated Au nanodrops to catalyze the etching of isolated pores. For this purpose, the non-close-packed Au nanodrops were used to form SiNPs on Si surface (Fig. 5b). The diameter of Au-generated pores varies from 250 up to 1 μm and corresponds to the size of Au nanodrops.

**Fig. 6:**
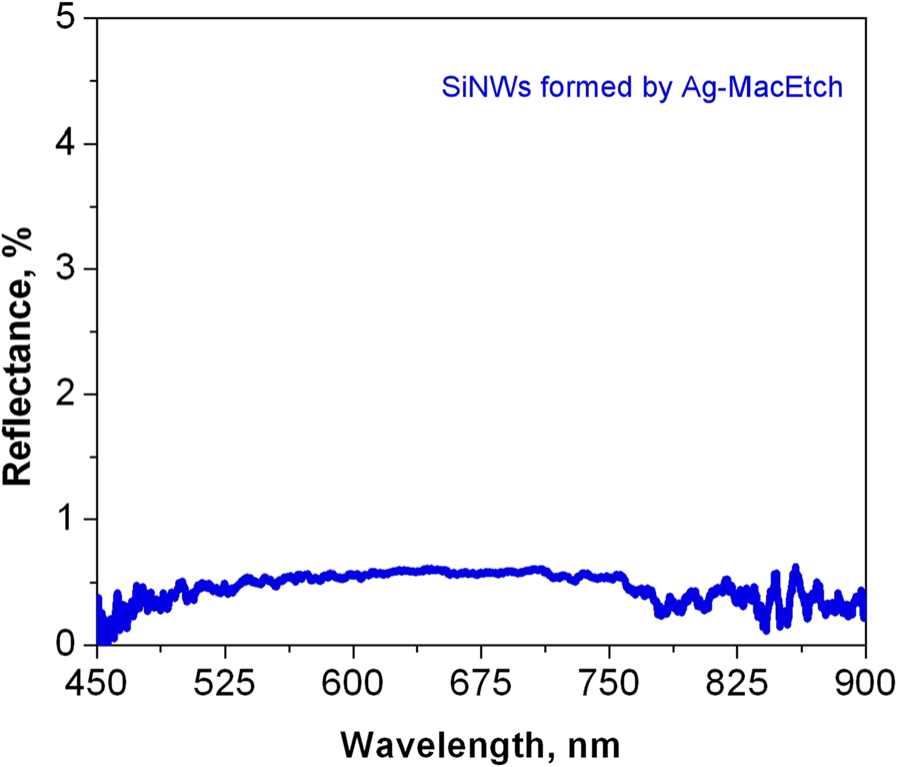
Reflectance spectrum of the SiNW arrays prepared by Ag-MacEtch in HF/H_2_O_2_ (4.6/0.15 M) solution

**Fig. 7:**
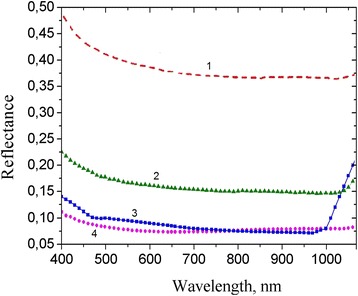
Reflectance as a function of wavelength: 1, clean Si wafer; 2, Si wafer with SiNPs produced by Au-MacEtch; 3, Si wafer with SiNWs produced by Ag-MacEtch; 4, Si wafer with SiNWs produced by Au-MacEtch

Figure 6 depicts the reflectance spectrum of the SiNW arrays prepared by Ag-MacEtch. As can be seen, the reflectance of 1% is observed mainly in the visible spectrum wavelengths. Figure 7 compares the optical reflection between clean Si wafer (served as reference), SiNWs, and SiNPs produced by Au-MacEtch and SiNWs produced by Ag-assisted MacEtch. As shown in Fig. 7 (curve 4), it is obvious that the reflectance of SiNWs produced by Au-MacEtch is as low as 1~5%, whereas, for Si samples with SiNPs produced by Au-MacEtch, this value corresponds to 17% (see Fig. 7, curve 2). At the same time, SiNWs produced by Ag-assisted MacEtch are characterized by lower reflectance (see Fig. 7, curve 3), which is comparable to those produced by Au-MacEtch. These results are in good agreement with previous findings that the use of longer SiNWs can result in a lower optical specular reflectance [31].

**Fig. 8:**
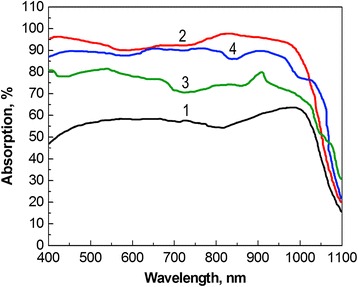
Absorption as a function of wavelength: 1, clean Si wafer; 2, Si wafer with SiNWs produced by Au-MacEtch; 3, Si wafer with SiNPs produced by Au-MacEtch; 4, Si wafer with SiNWs produced by Ag-MacEtch

In addition, an excelent light absorption of ~95–98% in the wavelength region above ~750 nm for the case of SiNWs produced by Au-MacEtch is shown in Fig. 8, curve 2. The similar was observed for SiNWs produced by Ag-MacEtch (Fig. 8, curve 4). The curve 3 in Fig. 8 corresponds to the absorption of SiNPs produced by Au-MacEtch, which is found to be less than the absorption of SiNWs, but naturally higher than that of clean Si wafer (Fig. 8, curve 1).

The aforementioned observations support the theory proposed by Li et al. [32]. It states that from the point of view of wave optics, the light wavelength in the low energy region (corresponds to long wavelengths) is much longer than the distance between the SiNW arrays. Accordingly, the incident light wave can easily penetrate through the SiNW array, reaching the underlying Si layer and interacting with it. This is well evidenced by the reflectance and absorption spectra of SiNW samples in the corresponding energy region. This also explains the higher light absorption of Au-MacEtch-ed SiNWs, the density of which is quite high, in comparison to Ag-produced SiNWs with lower density and partial size distribution. Moreover, the 5-μm-long SiNWs prepared by Au-MacEtch demonstrate better antireflective properties in contrast to the 2-μm-long SiNWs formed by conventional Ag-assisted chemical etching.

## Conclusions

In conclusion, metal-assisted Si chemical etching was performed using different approaches to form SiNW and SiNP arrays. The first one consisting of a deposition of Ag nanoparticles on Si substrate from AgNO_3_/HF solution and subsequent etching in HF/H_2_O_2_ resulted in the formation of 2-μm-long vertically aligned Si nanowires with diameters ranging from 64 to 240 nm. The second one included such steps as the thermal vacuum deposition of an Au thin film on Si substrate, annealing of these samples for the coagulation of an Au film into the nanodrops, and subsequent etching of as-prepared samples in HF/H_2_O_2_/H_2_O. By varying the annealing time, two patterns from close-packed and non-close-packed Au nanodrops were obtained on the Si surface. From these patterns, the 5-μm-long SiNWs and SiNPs with various diameters were formed in the etching process.

We investigated the influence of modified Si MacEtch technique on the morphologies and optical properties of Si substrate surface decorated with SiNWs and NPs to achieve the desirable antireflection for practical solar cell applications. Around 1~7% and ~17% of total surface reflectance were observed over the entire Si-absorbing region for the case of SiNWs and SiNPs, respectively. Meanwhile, 5-μm-long SiNWs fabricated by Au-MacEtch exhibited high absorption of 98% in the visible region of the spectrum. Therefore, the use of SiNWs obtained by the modified Au-MacEtch method as the antireflective material is favored over those prepared by Ag-MacEtch due to their higher light absorption and lower reflectance.

## Abbreviations

CVD: Chemical vapour deposition
DI: Deionized
MacEtch: Metal-assisted chemical etching
ND: Nanodrop
NP: Nanopore
NW: Nanowire
RCA: Radio Corporation of America
SC: Solar cell
SEM: Scanning electron microscope
Si: Silicon
